# Crystal structure of ethyl 2-methyl-5,10-dioxo-4-phenyl-5,10-di­hydro-4*H*-11-thia-1,4a-di­aza­benzo[*b*]fluorene-3-carb­oxy­late

**DOI:** 10.1107/S2056989018000117

**Published:** 2018-01-09

**Authors:** Yegor Yartsev, Pavel Lyubashov, Vyacheslav Povstyanoy, Mykhailo Povstyaniy, Iryna Lebedyeva

**Affiliations:** a Department of Inorganic Chemistry, V. N. Karazin Kharkiv National University, 4, Svobody sq., Kharkiv 61077, Ukraine; b Department of Chemical Technology and Food Safety, Kherson National Technical, University, Berislavs’ke Highway 24, Kherson 73008, Ukraine; c Department of Chemistry and Physics, Augusta University, 1120 15th Street, Augusta 30912, USA

**Keywords:** crystal structure, di­hydro­pyrimidine, enanti­omer, di­hydro­pyrimidine, DHPM

## Abstract

The title compound, C_24_H_18_N_2_O_4_S, crystallizes in the ortho­rhom­bic *P*2_1_2_1_2_1_ space group, indicating the existence of only one enanti­omer with an *S* configuration of the chiral center in the crystal phase. The di­hydro­pyrimidine ring adopts a twist-boat conformation while the quinone ring is slightly non-planar. In the crystal, mol­ecules are linked by weak C—H⋯O and C—H⋯S hydrogen bonds and C—H⋯π inter­actions. In addition, a short inter­molecular S⋯N contact of 3.250 (3) Å indicates an inter­action between the S atom and the π-system of the thia­zole ring.

## Abstract 2

The di­hydro­pyrimidine ring adopts a twist-boat conformation while the quinone ring is slightly non-planar. In the crystal, mol­ecules are linked by weak C—H⋯O and C—H⋯S hydrogen bonds and C—H⋯π inter­actions. In addition, a short inter­molecular S⋯N contact occurs.

## Chemical context   

**Figure 1:**
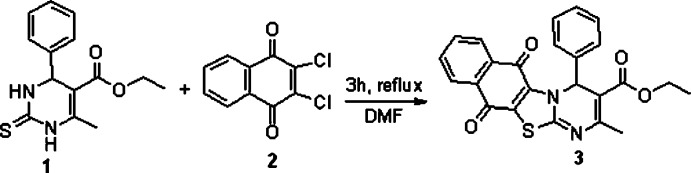
Synthesis of **3**.

The three-component Biginelli reaction allows the assembly of a wide variety of di­hydro­pyrimidine (DHPM) compounds that can be modified easily depending on the starting materials used during the reaction (Nagarajaiah *et al.*, 2016 ▸). DHPMs exhibit anti­bacterial (Wani *et al.*, 2017 ▸) and anti­fungal properties (Akhaja & Raval, 2012 ▸) and their thio­analogues such as monastrol are being used as inhibitors of mitotic kinesin Eg5 in the treatment of breast and ovarian tumors (Bobylev *et al.*, 2017 ▸; Duan *et al.*, 2015 ▸). In this work we investigated reactivity of thioDHPMs **1** in their reaction with the di-halogen-substituted nucleophile **2**. It was expected that the reaction would proceed with substitution of one or both chlorine atoms and the formation of a thia­zole ring in the product **3** (Fig. 1 ▸).
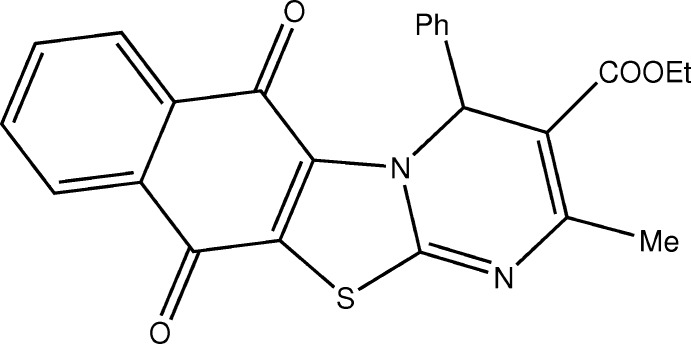


## Structural commentary   

**Figure 2:**
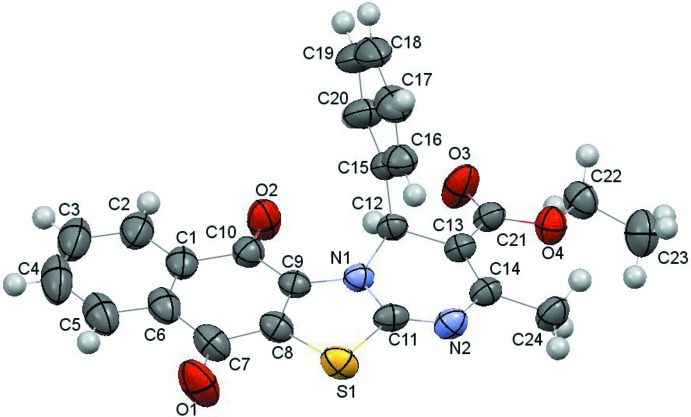
The mol­ecular structure of compound **3** with the atom labelling. Displacement ellipsoids are drawn at the 50% probability level.

Compound **3** crystallizes in the non-centrosymmetric chiral space group *P*2_1_2_1_2_1_. This indicates the existence of only one enanti­omer in the crystal with an *S*-configuration of the C12 chiral center according to the Flack parameter (Parsons *et al.*, 2013 ▸). The quinone ring is slightly non-planar (Fig. 2 ▸). Its conformation can be described as a flattened asymmetric screw-boat with the C7 and C10 atoms deviating by 0.053 (3) and 0.082 (3) Å from the mean plane through the remaining ring atoms. This non-planarity may be caused by the formation of the weak intra­molecular C12—H12⋯O2 hydrogen bond. Taking into account high conformational flexibility of the quinone ring (Shishkin, 1997 ▸; Kovalevsky *et al.*, 1998 ▸), it could be suggested that the out-of plane deformation of the ring represents the easiest way for relaxation of its structure because of steric clashes.

The di­hydro­pyrimidine ring adopts a twist-boat conformation with puckering parameters (Zefirov *et al.*, 1990 ▸) *S* = 0.34, Θ = 61.5° and ψ = 12.5°. The deviations of the C11 and N1 atoms from the mean plane through the remaining ring atoms are 0.307 (2) and 0.458 (2) Å, respectively. Such a conformation is common for 1,6-di­hydro­aromatic heterocycles (Shishkin, 1998 ▸). However, the presence of three vicinal substituents results in some twisting of the N2—C11 and C13—C14 endocyclic double bonds [the C14—N2—C11—N1 and C12—C13—C14—N2 torsion angles are −7.6 (4) and 4.5 (4)°] because of steric repulsion [the intra­molecular O4⋯C24 contact is 2.739 (4) Å, shorter than the sum of the van der Waals radii (2.87 Å; Zefirov, 1997 ▸). The phenyl substituent has an axial orientation with respect to the di­hydro­pyrimidine ring [C11—N1—C12—C15 = −98.7 (3)°] and is almost coplanar with the C12—H12 bond (C20—C15—C12—H12 = −8°) despite the shorten intra­molecular H12⋯H20 contact of 2.30 Å (sum of van der Waals radii = 2.32 Å). The carbonyl group of the ester substituent is rotated slightly with respect to the C13—C14 bond [C14—C13—C21—O3 = 167.2 (2)°] probably as a result of the formation of the O3⋯H12 attractive intra­molecular inter­action (2.34 Å compared to the van der Waals radii sum of 2.45 Å). The ethoxyl group has an *ap*–*ap* conformation [C13—C21—O4—C22 and C21—O4—C22—C23 torsion angles are 175.1 (2) and −160.5 (3)°, respectively].

## Supra­molecular features   

**Figure 3:**
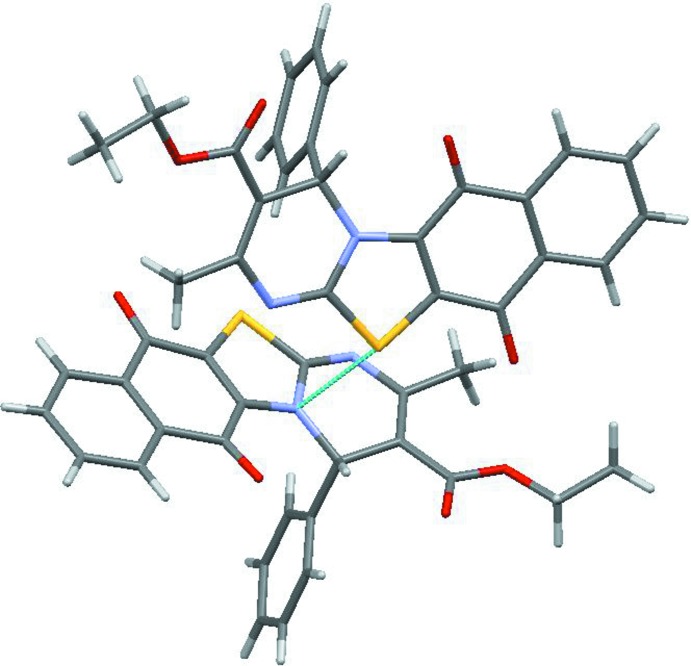
The view of the S⋯N inter­molecular inter­action.

In the crystal, mol­ecules are linked by weak C19—H19⋯O3 and C16—H16⋯S1 hydrogen bonds and C—H⋯π inter­actions (C3—H3⋯C16 and C22—H22*A*⋯C2 (see Table 1 ▸). In addition, a short inter­molecular S1⋯N1(−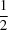 + *x*, 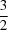 − *y*, 2 − *z*) contact of 3.250 (3) Å (van der Waals radii sum is 3.32 Å) indicates an inter­action between the S atom and the π-system of the thia­zole ring (Fig. 3 ▸).

## Database survey   

A search of the Cambridge Structural Database (Version 5.37, update May 2016; Groom *et al.*, 2016 ▸) did not reveal any compounds with a similar polycyclic fragment.

## Synthesis and crystallization   

Ethyl 6-methyl-4-phenyl-2-thioxo-1,2,3,4-tetra­hydro­pyrim­id­ine-5-carboxyl­ate **1** (0.28 g, 1 mmol) was added to a solution of 2,3-di­chloro­naphthalene-1,4-dione (0.25 g, 1.1 mmol) in DMF (20 mL) and the mixture was kept under reflux for 3 h. After that, the reaction mixture was cooled, and the precipitated solid product was filtered off and purified *via* recrystallization from MeOH:DMF:H_2_O (2:2:1) to give product **3** in the form of dark-red crystals in 78% yield (0.35 g), m.p. 520.3– 522.0 K.

## Refinement   

Crystal data, data collection and structure refinement details are summarized in Table 2 ▸. All H atoms were located in difference-Fourier maps and treated as riding (C—H = 0.93–0.97 Å) with *U*_iso_(H) = *nU*_eq_(C) (*n* = 1.5 for CH_3_ and *n* = 1.2 for all other H atoms).

## Supplementary Material

Crystal structure: contains datablock(s) I. DOI: 10.1107/S2056989018000117/zq2240sup1.cif

Structure factors: contains datablock(s) I. DOI: 10.1107/S2056989018000117/zq2240Isup2.hkl

CCDC reference: 1814348

Additional supporting information:  crystallographic information; 3D view; checkCIF report

## Figures and Tables

**Table 1:** Hydrogen-bond geometry (Å, °)

*D*—H⋯*A*	*D*—H	H⋯*A*	*D*⋯*A*	*D*—H⋯*A*
C12—H12⋯O2	0.98	2.36	2.926 (4)	116
C19—H19⋯O3^i^	0.93	2.50	3.219 (5)	134
C16—H16⋯S1^ii^	0.93	3.07	3.810 (4)	138
C3—H3⋯C16^iii^	0.93	2.84	3.542 (5)	133
C22—H22*A*⋯C2^iv^	0.97	2.88	3.718 (5)	145

Symmetry codes: (i) 
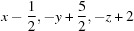
; (ii) 
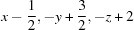
; (iii) 
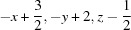
; (iv) 
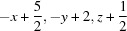
.

**Table 2:** Experimental details

Crystal data	
Chemical formula	C_24_H_18_N_2_O_4_S
*M*_r_	430.46
Crystal system, space group	Orthorhombic, *P*2_1_2_1_2_1_
Temperature (K)	293
*a*, *b*, *c* (Å)	8.1038 (3), 13.3915 (6), 18.7377 (9)
*V* (Å^3^)	2033.44 (15)
*Z*	4
Radiation type	Mo *K*α
μ (mm^−1^)	0.19
Crystal size (mm)	0.14 × 0.14 × 0.12

Data collection	
Diffractometer	Oxford Diffraction Xcalibur, Sapphire3
Absorption correction	Multi-scan (*CrysAlis PRO*; Oxford Diffraction, 2010 ▸
*T*_min_, *T*_max_	0.991, 1.000
No. of measured, independent and observed [*I* > 2σ(*I*)] reflections	6202, 3574, 3160
*R*_int_	0.017
(sin θ/λ)_max_ (Å^−1^)	0.594

Refinement	
*R*[*F* ^2^ > 2σ(*F* ^2^)], *wR*(*F* ^2^), *S*	0.035, 0.087, 1.03
No. of reflections	3574
No. of parameters	282
H-atom treatment	H-atom parameters constrained
Δρ_max_, Δρ_min_ (e Å^−3^)	0.13, −0.14
Absolute structure	Flack *x* determined using 1207 quotients [(*I* ^+^)−(*I* ^−^)]/[(*I* ^+^)+(*I* ^−^)] (Parsons *et al.*, 2013 ▸).
Absolute structure parameter	0.00 (4)

Computer programs: (*CrysAlis PRO*; Oxford Diffraction, 2010 ▸, *SHELXT* (Sheldrick, 2015*a* ▸), *SHELXL2014/7* (Sheldrick, 2015*b* ▸), *Mercury* (Macrae *et al.*, 2008 ▸), *publCIF* (Westrip, 2010 ▸).

## supplementary crystallographic information

### Crystal data

**Table:**

C_24_H_18_N_2_O_4_S	*D*_x_ = 1.406 Mg m^−^^3^
*M**_r_* = 430.46	Mo *K*α radiation, λ = 0.71073 Å
Orthorhombic, *P*2_1_2_1_2_1_	Cell parameters from 3614 reflections
*a* = 8.1038 (3) Å	θ = 3.0–29.0°
*b* = 13.3915 (6) Å	µ = 0.19 mm^−^^1^
*c* = 18.7377 (9) Å	*T* = 293 K
*V* = 2033.44 (15) Å^3^	Block, red
*Z* = 4	0.14 × 0.14 × 0.12 mm
*F*(000) = 896	

### Data collection

**Table:**

Oxford Diffraction Xcalibur, Sapphire3 diffractometer	3574 independent reflections
Radiation source: Enhance (Mo) X-ray Source	3160 reflections with *I* > 2σ(*I*)
Detector resolution: 16.1827 pixels mm^-1^	*R*_int_ = 0.017
ω–scan	θ_max_ = 25.0°, θ_min_ = 3.0°
Absorption correction: multi-scan (CrysAlisPro; Oxford Diffraction, 2010	*h* = −9→9
*T*_min_ = 0.991, *T*_max_ = 1.000	*k* = −13→15
6202 measured reflections	*l* = −22→22

### Refinement

**Table:**

Refinement on *F*^2^	Secondary atom site location: difference Fourier map
Least-squares matrix: full	Hydrogen site location: inferred from neighbouring sites
*R*[*F*^2^ > 2σ(*F*^2^)] = 0.035	H-atom parameters constrained
*wR*(*F*^2^) = 0.087	*w* = 1/[σ^2^(*F*_o_^2^) + (0.0436*P*)^2^ + 0.1932*P*] where *P* = (*F*_o_^2^ + 2*F*_c_^2^)/3
*S* = 1.03	(Δ/σ)_max_ < 0.001
3574 reflections	Δρ_max_ = 0.13 e Å^−^^3^
282 parameters	Δρ_min_ = −0.14 e Å^−^^3^
0 restraints	Absolute structure: Flack *x* determined using 1207 quotients [(*I*^+^)-(*I*^-^)]/[(*I*^+^)+(*I*^-^)] (Parsons *et al.*, 2013).
Primary atom site location: difference Fourier map	Absolute structure parameter: 0.00 (4)

### Special details

**Table:**

Geometry. All esds (except the esd in the dihedral angle between two l.s. planes) are estimated using the full covariance matrix. The cell esds are taken into account individually in the estimation of esds in distances, angles and torsion angles; correlations between esds in cell parameters are only used when they are defined by crystal symmetry. An approximate (isotropic) treatment of cell esds is used for estimating esds involving l.s. planes.

### Fractional atomic coordinates and isotropic or equivalent isotropic displacement parameters (Å^2^)

**Table:**

	*x*	*y*	*z*	*U*_iso_*/*U*_eq_	
S1	0.70278 (10)	0.68425 (6)	0.96988 (5)	0.0577 (2)	
O1	0.7487 (4)	0.6165 (2)	0.81545 (17)	0.0925 (9)	
O2	1.0119 (3)	0.97193 (18)	0.87923 (13)	0.0727 (7)	
O3	1.0162 (4)	1.08986 (18)	1.09805 (13)	0.0801 (8)	
O4	0.9283 (2)	1.03242 (17)	1.20226 (11)	0.0561 (6)	
N1	0.8225 (3)	0.86059 (16)	0.98532 (12)	0.0414 (5)	
N2	0.7164 (3)	0.78761 (17)	1.09161 (14)	0.0499 (6)	
C1	0.9415 (4)	0.8572 (3)	0.78826 (16)	0.0527 (8)	
C2	1.0157 (4)	0.9143 (3)	0.73617 (18)	0.0670 (10)	
H2	1.0638	0.9752	0.7478	0.080*	
C3	1.0179 (5)	0.8804 (4)	0.6664 (2)	0.0827 (12)	
H3	1.0673	0.9191	0.6311	0.099*	
C4	0.9489 (5)	0.7914 (4)	0.6487 (2)	0.0890 (14)	
H4	0.9493	0.7704	0.6014	0.107*	
C5	0.8785 (5)	0.7318 (4)	0.7004 (2)	0.0766 (11)	
H5	0.8338	0.6701	0.6881	0.092*	
C6	0.8745 (4)	0.7644 (3)	0.77107 (18)	0.0589 (9)	
C7	0.8000 (4)	0.7003 (3)	0.82700 (19)	0.0604 (8)	
C8	0.7887 (4)	0.7446 (2)	0.89746 (17)	0.0497 (7)	
C9	0.8483 (3)	0.8355 (2)	0.91469 (16)	0.0428 (7)	
C10	0.9400 (4)	0.8963 (2)	0.86233 (17)	0.0499 (7)	
C11	0.7472 (3)	0.7863 (2)	1.02436 (18)	0.0450 (7)	
C12	0.8429 (3)	0.96026 (19)	1.01698 (15)	0.0415 (6)	
H12	0.9494	0.9872	1.0014	0.050*	
C13	0.8477 (3)	0.9481 (2)	1.09764 (15)	0.0415 (6)	
C14	0.7823 (3)	0.8678 (2)	1.13005 (16)	0.0446 (7)	
C15	0.7081 (4)	1.0300 (2)	0.99115 (14)	0.0438 (6)	
C16	0.5438 (4)	1.0063 (3)	0.99771 (17)	0.0565 (8)	
H16	0.5133	0.9456	1.0179	0.068*	
C17	0.4239 (5)	1.0717 (3)	0.9747 (2)	0.0722 (10)	
H17	0.3131	1.0548	0.9792	0.087*	
C18	0.4674 (6)	1.1617 (3)	0.9451 (2)	0.0774 (11)	
H18	0.3864	1.2060	0.9298	0.093*	
C19	0.6310 (6)	1.1857 (3)	0.9383 (2)	0.0832 (12)	
H19	0.6611	1.2466	0.9183	0.100*	
C20	0.7504 (5)	1.1206 (2)	0.9608 (2)	0.0660 (9)	
H20	0.8611	1.1374	0.9556	0.079*	
C21	0.9387 (4)	1.0299 (2)	1.13135 (16)	0.0471 (7)	
C22	1.0268 (4)	1.1067 (3)	1.23806 (18)	0.0609 (9)	
H22B	0.9735	1.1714	1.2355	0.073*	
H22A	1.1346	1.1117	1.2158	0.073*	
C23	1.0436 (5)	1.0742 (3)	1.3141 (2)	0.0815 (12)	
H23B	0.9359	1.0665	1.3348	0.122*	
H23C	1.1043	1.1236	1.3403	0.122*	
H23A	1.1012	1.0116	1.3160	0.122*	
C24	0.7765 (4)	0.8499 (2)	1.20923 (17)	0.0594 (8)	
H24C	0.8858	0.8554	1.2287	0.089*	
H24A	0.7341	0.7842	1.2184	0.089*	
H24B	0.7061	0.8986	1.2312	0.089*	

### Atomic displacement parameters (Å^2^)

**Table:**

	*U*^11^	*U*^22^	*U*^33^	*U*^12^	*U*^13^	*U*^23^
S1	0.0570 (4)	0.0437 (4)	0.0725 (5)	−0.0110 (4)	0.0073 (4)	−0.0060 (4)
O1	0.105 (2)	0.0743 (17)	0.098 (2)	−0.0213 (16)	0.0155 (17)	−0.0383 (17)
O2	0.0955 (17)	0.0606 (14)	0.0620 (14)	−0.0237 (14)	0.0224 (14)	−0.0034 (13)
O3	0.121 (2)	0.0638 (14)	0.0554 (13)	−0.0474 (16)	−0.0102 (15)	0.0097 (13)
O4	0.0569 (13)	0.0627 (13)	0.0486 (11)	−0.0122 (11)	−0.0002 (10)	−0.0033 (11)
N1	0.0428 (12)	0.0360 (12)	0.0452 (13)	−0.0025 (10)	0.0018 (11)	0.0033 (11)
N2	0.0532 (14)	0.0423 (13)	0.0542 (15)	−0.0095 (12)	0.0019 (13)	0.0078 (12)
C1	0.0458 (16)	0.064 (2)	0.0482 (17)	0.0134 (15)	−0.0009 (14)	−0.0004 (16)
C2	0.065 (2)	0.082 (3)	0.0540 (19)	0.0140 (19)	0.0023 (18)	0.007 (2)
C3	0.082 (3)	0.115 (4)	0.051 (2)	0.020 (3)	0.001 (2)	0.009 (2)
C4	0.080 (3)	0.140 (4)	0.047 (2)	0.022 (3)	−0.003 (2)	−0.012 (3)
C5	0.069 (2)	0.096 (3)	0.065 (2)	0.015 (2)	−0.008 (2)	−0.024 (2)
C6	0.0482 (17)	0.073 (2)	0.0555 (19)	0.0138 (16)	−0.0033 (16)	−0.0110 (18)
C7	0.0503 (16)	0.060 (2)	0.071 (2)	0.0030 (17)	0.0007 (17)	−0.0185 (18)
C8	0.0419 (15)	0.0473 (16)	0.0598 (18)	0.0009 (14)	0.0013 (15)	−0.0053 (15)
C9	0.0374 (13)	0.0410 (16)	0.0501 (16)	0.0042 (11)	−0.0016 (12)	−0.0006 (14)
C10	0.0487 (17)	0.0497 (18)	0.0514 (17)	0.0037 (15)	0.0036 (14)	0.0034 (15)
C11	0.0373 (14)	0.0371 (15)	0.0605 (19)	−0.0037 (11)	0.0011 (13)	0.0068 (15)
C12	0.0478 (15)	0.0309 (13)	0.0458 (15)	−0.0033 (11)	0.0024 (12)	0.0032 (13)
C13	0.0421 (14)	0.0383 (14)	0.0441 (15)	−0.0003 (12)	−0.0025 (12)	0.0044 (13)
C14	0.0417 (14)	0.0446 (15)	0.0475 (16)	−0.0003 (13)	0.0005 (14)	0.0079 (14)
C15	0.0537 (16)	0.0373 (15)	0.0405 (14)	0.0042 (13)	−0.0029 (13)	0.0013 (12)
C16	0.0561 (18)	0.0533 (17)	0.0600 (18)	0.0068 (15)	−0.0048 (15)	0.0014 (16)
C17	0.063 (2)	0.081 (3)	0.072 (2)	0.0174 (19)	−0.014 (2)	−0.007 (2)
C18	0.099 (3)	0.068 (2)	0.065 (2)	0.037 (2)	−0.022 (2)	−0.001 (2)
C19	0.107 (3)	0.056 (2)	0.087 (3)	0.019 (2)	−0.009 (3)	0.020 (2)
C20	0.074 (2)	0.0463 (18)	0.078 (2)	0.0044 (15)	−0.0003 (19)	0.0149 (18)
C21	0.0526 (16)	0.0405 (16)	0.0482 (16)	−0.0021 (14)	−0.0042 (14)	0.0065 (15)
C22	0.062 (2)	0.062 (2)	0.0587 (19)	−0.0050 (17)	−0.0081 (17)	−0.0109 (17)
C23	0.085 (3)	0.101 (3)	0.058 (2)	−0.009 (2)	−0.004 (2)	−0.010 (2)
C24	0.071 (2)	0.0556 (18)	0.0518 (18)	−0.0078 (16)	0.0021 (17)	0.0139 (16)

### Geometric parameters (Å, º)

**Table:**

S1—C8	1.726 (3)	C12—C15	1.517 (4)
S1—C11	1.743 (3)	C12—C13	1.521 (4)
O1—C7	1.216 (4)	C12—H12	0.9800
O2—C10	1.211 (4)	C13—C14	1.344 (4)
O3—C21	1.195 (3)	C13—C21	1.463 (4)
O4—C21	1.332 (4)	C14—C24	1.504 (4)
O4—C22	1.441 (4)	C15—C16	1.374 (4)
N1—C11	1.378 (3)	C15—C20	1.383 (4)
N1—C9	1.381 (4)	C16—C17	1.377 (4)
N1—C12	1.470 (4)	C16—H16	0.9300
N2—C11	1.285 (4)	C17—C18	1.373 (6)
N2—C14	1.399 (4)	C17—H17	0.9300
C1—C2	1.378 (5)	C18—C19	1.371 (6)
C1—C6	1.394 (5)	C18—H18	0.9300
C1—C10	1.484 (4)	C19—C20	1.369 (5)
C2—C3	1.384 (5)	C19—H19	0.9300
C2—H2	0.9300	C20—H20	0.9300
C3—C4	1.358 (6)	C22—C23	1.497 (5)
C3—H3	0.9300	C22—H22B	0.9700
C4—C5	1.378 (6)	C22—H22A	0.9700
C4—H4	0.9300	C23—H23B	0.9600
C5—C6	1.395 (5)	C23—H23C	0.9600
C5—H5	0.9300	C23—H23A	0.9600
C6—C7	1.483 (5)	C24—H24C	0.9600
C7—C8	1.451 (5)	C24—H24A	0.9600
C8—C9	1.348 (4)	C24—H24B	0.9600
C9—C10	1.476 (4)		
			
C8—S1—C11	90.57 (14)	C14—C13—C21	127.1 (3)
C21—O4—C22	116.5 (2)	C14—C13—C12	121.6 (3)
C11—N1—C9	113.6 (2)	C21—C13—C12	111.2 (2)
C11—N1—C12	119.4 (2)	C13—C14—N2	122.2 (3)
C9—N1—C12	126.2 (2)	C13—C14—C24	125.9 (3)
C11—N2—C14	116.2 (2)	N2—C14—C24	111.9 (3)
C2—C1—C6	120.1 (3)	C16—C15—C20	118.7 (3)
C2—C1—C10	118.0 (3)	C16—C15—C12	121.8 (3)
C6—C1—C10	121.9 (3)	C20—C15—C12	119.5 (3)
C1—C2—C3	119.5 (4)	C15—C16—C17	120.6 (3)
C1—C2—H2	120.3	C15—C16—H16	119.7
C3—C2—H2	120.3	C17—C16—H16	119.7
C4—C3—C2	120.8 (4)	C18—C17—C16	120.2 (4)
C4—C3—H3	119.6	C18—C17—H17	119.9
C2—C3—H3	119.6	C16—C17—H17	119.9
C3—C4—C5	120.5 (4)	C19—C18—C17	119.5 (3)
C3—C4—H4	119.7	C19—C18—H18	120.3
C5—C4—H4	119.7	C17—C18—H18	120.3
C4—C5—C6	119.7 (4)	C20—C19—C18	120.4 (4)
C4—C5—H5	120.2	C20—C19—H19	119.8
C6—C5—H5	120.2	C18—C19—H19	119.8
C1—C6—C5	119.3 (4)	C19—C20—C15	120.7 (4)
C1—C6—C7	120.7 (3)	C19—C20—H20	119.7
C5—C6—C7	120.0 (4)	C15—C20—H20	119.7
O1—C7—C8	121.2 (3)	O3—C21—O4	122.5 (3)
O1—C7—C6	123.2 (3)	O3—C21—C13	122.8 (3)
C8—C7—C6	115.6 (3)	O4—C21—C13	114.7 (3)
C9—C8—C7	124.4 (3)	O4—C22—C23	107.0 (3)
C9—C8—S1	112.3 (2)	O4—C22—H22B	110.3
C7—C8—S1	123.3 (2)	C23—C22—H22B	110.3
C8—C9—N1	113.3 (3)	O4—C22—H22A	110.3
C8—C9—C10	121.3 (3)	C23—C22—H22A	110.3
N1—C9—C10	125.4 (3)	H22B—C22—H22A	108.6
O2—C10—C9	122.0 (3)	C22—C23—H23B	109.5
O2—C10—C1	122.4 (3)	C22—C23—H23C	109.5
C9—C10—C1	115.5 (3)	H23B—C23—H23C	109.5
N2—C11—N1	126.6 (3)	C22—C23—H23A	109.5
N2—C11—S1	123.1 (2)	H23B—C23—H23A	109.5
N1—C11—S1	110.3 (2)	H23C—C23—H23A	109.5
N1—C12—C15	110.4 (2)	C14—C24—H24C	109.5
N1—C12—C13	107.9 (2)	C14—C24—H24A	109.5
C15—C12—C13	113.7 (2)	H24C—C24—H24A	109.5
N1—C12—H12	108.2	C14—C24—H24B	109.5
C15—C12—H12	108.2	H24C—C24—H24B	109.5
C13—C12—H12	108.2	H24A—C24—H24B	109.5
			
C6—C1—C2—C3	2.3 (5)	C9—N1—C11—N2	176.2 (3)
C10—C1—C2—C3	−179.4 (3)	C12—N1—C11—N2	−13.3 (4)
C1—C2—C3—C4	−0.4 (6)	C9—N1—C11—S1	−1.8 (3)
C2—C3—C4—C5	−1.5 (6)	C12—N1—C11—S1	168.65 (19)
C3—C4—C5—C6	1.6 (6)	C8—S1—C11—N2	−177.5 (3)
C2—C1—C6—C5	−2.2 (5)	C8—S1—C11—N1	0.6 (2)
C10—C1—C6—C5	179.6 (3)	C11—N1—C12—C15	−98.7 (3)
C2—C1—C6—C7	177.6 (3)	C9—N1—C12—C15	70.4 (3)
C10—C1—C6—C7	−0.6 (4)	C11—N1—C12—C13	26.1 (3)
C4—C5—C6—C1	0.3 (5)	C9—N1—C12—C13	−164.8 (2)
C4—C5—C6—C7	−179.5 (3)	N1—C12—C13—C14	−22.3 (4)
C1—C6—C7—O1	−175.6 (3)	C15—C12—C13—C14	100.5 (3)
C5—C6—C7—O1	4.2 (5)	N1—C12—C13—C21	154.3 (2)
C1—C6—C7—C8	5.5 (4)	C15—C12—C13—C21	−82.9 (3)
C5—C6—C7—C8	−174.7 (3)	C21—C13—C14—N2	−171.5 (3)
O1—C7—C8—C9	177.5 (3)	C12—C13—C14—N2	4.5 (4)
C6—C7—C8—C9	−3.6 (5)	C21—C13—C14—C24	5.7 (5)
O1—C7—C8—S1	−0.2 (5)	C12—C13—C14—C24	−178.3 (3)
C6—C7—C8—S1	178.7 (2)	C11—N2—C14—C13	11.8 (4)
C11—S1—C8—C9	0.7 (2)	C11—N2—C14—C24	−165.7 (3)
C11—S1—C8—C7	178.6 (3)	N1—C12—C15—C16	54.1 (4)
C7—C8—C9—N1	−179.8 (3)	C13—C12—C15—C16	−67.3 (4)
S1—C8—C9—N1	−1.9 (3)	N1—C12—C15—C20	−126.6 (3)
C7—C8—C9—C10	−3.3 (4)	C13—C12—C15—C20	112.0 (3)
S1—C8—C9—C10	174.6 (2)	C20—C15—C16—C17	−0.2 (5)
C11—N1—C9—C8	2.4 (3)	C12—C15—C16—C17	179.1 (3)
C12—N1—C9—C8	−167.3 (3)	C15—C16—C17—C18	−0.2 (5)
C11—N1—C9—C10	−173.9 (2)	C16—C17—C18—C19	0.4 (6)
C12—N1—C9—C10	16.4 (4)	C17—C18—C19—C20	0.0 (7)
C8—C9—C10—O2	−170.5 (3)	C18—C19—C20—C15	−0.4 (6)
N1—C9—C10—O2	5.5 (5)	C16—C15—C20—C19	0.5 (5)
C8—C9—C10—C1	8.0 (4)	C12—C15—C20—C19	−178.8 (3)
N1—C9—C10—C1	−175.9 (3)	C22—O4—C21—O3	−5.1 (5)
C2—C1—C10—O2	−5.8 (5)	C22—O4—C21—C13	175.1 (2)
C6—C1—C10—O2	172.5 (3)	C14—C13—C21—O3	167.2 (3)
C2—C1—C10—C9	175.7 (3)	C12—C13—C21—O3	−9.1 (4)
C6—C1—C10—C9	−6.1 (4)	C14—C13—C21—O4	−12.9 (4)
C14—N2—C11—N1	−7.6 (4)	C12—C13—C21—O4	170.8 (2)
C14—N2—C11—S1	170.2 (2)	C21—O4—C22—C23	−160.5 (3)

### Hydrogen-bond geometry (Å, º)

**Table:**

*D*—H···*A*	*D*—H	H···*A*	*D*···*A*	*D*—H···*A*
C12—H12···O2	0.98	2.36	2.926 (4)	116
C19—H19···O3^i^	0.93	2.50	3.219 (5)	134
C16—H16···S1^ii^	0.93	3.07	3.810 (4)	138
C3—H3···C16^iii^	0.93	2.84	3.542 (5)	133
C22—H22*A*···C2^iv^	0.97	2.88	3.718 (5)	145

Symmetry codes: (i) *x*−1/2, −*y*+5/2, −*z*+2; (ii) *x*−1/2, −*y*+3/2, −*z*+2; (iii) −*x*+3/2, −*y*+2, *z*−1/2; (iv) −*x*+5/2, −*y*+2, *z*+1/2.

## References

[bb1] Akhaja, T. N. & Raval, J. P. (2012). *Chin. Chem. Lett.* **23**, 446–449.

[bb2] Bobylev, I., Peters, D., Vyas, M., Barham, M., Klein, I., von Strandmann, E. P., Neiss, W. F. & Lehmann, H. C. (2017). *Neurotox. Res.* **32**, 555–562. 10.1007/s12640-017-9760-7 28612296

[bb3] Duan, L., Wang, T.-Q., Bian, W., Liu, W., Sun, Y. & Yang, B.-S. (2015). *Spectrochim. Acta A Mol. Biomol. Spectrosc.* **137**, 1086–1091. 10.1016/j.saa.2014.08.050 25300040

[bb4] Groom, C. R., Bruno, I. J., Lightfoot, M. P. & Ward, S. C. (2016). *Acta Cryst.* B**72**, 171–179. 10.1107/S2052520616003954 PMC4822653 27048719

[bb5] Kovalevsky, A. Yu., Shishkin, O. V. & Dekaprilevich, M. O. (1998). *Russ. Chem. Bull.* **47**, 372–374.

[bb6] Macrae, C. F., Bruno, I. J., Chisholm, J. A., Edgington, P. R., McCabe, P., Pidcock, E., Rodriguez-Monge, L., Taylor, R., van de Streek, J. & Wood, P. A. (2008). *J. Appl. Cryst.* **41**, 466–470.

[bb7] Nagarajaiah, H., Mukhopadhyay, A. & Moorthy, J. N. (2016). *Tetrahedron Lett.* **57**, 5135–5149.

[bb8] Oxford Diffraction (2010). *CrysAlis PRO.* Oxford Diffraction Ltd, Yarnton, England.

[bb9] Parsons, S., Flack, H. D. & Wagner, T. (2013). *Acta Cryst.* B**69**, 249–259. 10.1107/S2052519213010014 PMC3661305 23719469

[bb11] Sheldrick, G. M. (2015*a*). *Acta Cryst.* A**71**, 3–8.

[bb12] Sheldrick, G. M. (2015*b*). *Acta Cryst.* C**71**, 3–8.

[bb13] Shishkin, O. V. (1997). *J. Mol. Struct.* **412**, 115–120.

[bb14] Shishkin, O. V. (1998). *J. Mol. Struct.* **447**, 217–222.

[bb15] Wani, M. Y., Ahmad, A., Kumar, S. & Sobral, A. J. F. N. (2017). *Microb. Pathog.* **105**, 57–62. 10.1016/j.micpath.2017.02.006 28189732

[bb16] Westrip, S. P. (2010). *J. Appl. Cryst.* **43**, 920–925.

[bb17] Zefirov, Yu. V. (1997). *Kristallografiya*, **42**, 936–958 (in Russian).

[bb18] Zefirov, N. S., Palyulin, V. A. & Dashevskaya, E. E. (1990). *J. Phys. Org. Chem.* **3**, 147–158.

